# A theoretical investigation of the effect of Ga alloying on thermodynamic stability, electronic-structure, and oxidation resistance of Ti_2_AlC MAX phase

**DOI:** 10.1038/s41598-022-17365-y

**Published:** 2022-07-29

**Authors:** Daniel Sauceda, Prashant Singh, Raymundo Arroyave

**Affiliations:** 1 Department of Materials Science and Engineering, Texas A&M University, College Station, TX 77843 USA; 2 Ames Laboratory, U.S. Department of Energy, Iowa State University, Ames, IA 50011 USA; 3 J. Mike Walker ’66 Department of Mechanical Engineering, Texas A&M University, College Station, TX 77843 USA; 4 Wm Michael Barnes ’64 Department of Industrial and Systems Engineering, Texas A&M University, College Station, TX 77843 USA

**Keywords:** Materials science, Theory and computation, Electronic structure

## Abstract

We present a systematic investigation of thermodynamic stability, phase-reaction, and chemical activity of Al containing disordered Ti_2_(Al-Ga)C MAX phases using machine-learning driven high-throughput framework to understand the oxidation resistance behavior with increasing temperature and exposure to static oxygen. The A-site (at Al) disordering in  Ti_2_AlC MAX (M=Ti, A=Al, X=C) with Ga shows significant change in the chemical activity of Al with increasing temperature and exposure to static oxygen, which is expected to enable surface segregation of Al, thereby, the formation of Al_2_O_3_ and improved oxidation resistance. We performed in-depth convex hull analysis of ternary Ti–Al–C, Ti–Ga–C, and Ti–Al–Ga–C based MAX phase, and provide detailed contribution arising from electronic, chemical and vibrational entropies. The thermodynamic analysis shows change in the Gibbs formation enthalpy (Δ*G*_form_) at higher temperatures, which implies an interplay of temperature-dependent enthalpy and entropic contributions in oxidation resistance Ga doped Ti_2_AlC MAX phases. A detailed electronic structure and chemical bonding analysis using crystal orbital Hamilton population method reveal the origin of change in phases stability and in oxidation resistance in disorder Ti_2_(Al_1−x_Ga_x_)C MAX phases. Our electronic structure analysis correlate well with the change in oxidation resistance of Ga doped MAX phases. We believe our study provides a useful guideline to understand to role of alloying on electronic, thermodynamic, and oxidation related mechanisms of bulk MAX phases, which can work as a precursor to understand oxidation behavior of two-dimensional MAX phases, i.e., MXenes (transition metal carbides, carbonitrides and nitrides).

## Introduction

MAX phases are a family of layered ternary compounds with hexagonal symmetry, which get their ceramic and metallic properties from the alternatively arranged covalently bonded (M–X) and metallic (M–A) layers^1–5^. The high strength, good damage tolerance, machinability, exceptional thermal shock resistance, elastic stiffness, and thermal/electrical conductivity are equivalent to metal like high-temperature mechanical response, and outstanding ceramics like oxidation and corrosion resistance. Accordingly, there is a great interest in new MAX phases with improved properties like oxidation resistance. However, good oxidation resistance in crystalline materials requires the formation of protective oxidation layers. In high-temperature applications, the oxide phase formation depends heavily on the differences in the preparation process and atomic content. The MAX phases with significant Al, Cr, or Si, present in Ti_2_AlC, Cr_2_AlC, or Ti_3_SiC_2_ MAX phases, are known to enable good oxidation resistance through the formation of protective oxide layers, e.g., Al_2_O_3_, Cr_2_O_3_, or SiO_2_, at high operating temperatures^6,7^. The atoms with metallic bonds in A-layer, e.g., Al/Cr/Si, are weaker compared to covalently bonded atoms in M (early transition metals)-X (group 13–16 element) layers. This results in smaller diffusion barriers for Al/Cr/Si atoms at A-sites, thereby, enabling the formation of protective oxides^6,8–13^.

Ti_2_AlC based MAX phases are projected as potential candidates for fuel coating and structural applications in nuclear (fusion and fission) reactors for their superior oxidation resistance properties^14–17^. Recently however, Sokol et al*.* has reported that Ti_2_AlC does not provide a suitable protective coating against oxidation, while Cr_2_AlC was found to produce a suitable oxidation barrier, i.e., an outstanding candidate^18^. Notably, the Ti_2_GaC is another candidate material with remarkable electronic (phase stability and electronic-structure), structural (stability of crystal phases), and mechanical (exceptional thermal shock resistance and damage tolerance, excellent oxidation resistance, and elastic stiffness) properties^1,19–27^. Several ab-initio calculated MAX phase properties were found in good agreement with experiments^28–32^, although high temperature studies of oxidation behavior of disordered Ti_2_AlC/Ti_2_GaC MAX phase are limited^33^. Therefore, it remains a challenge to understand the role of disorder and temperature on thermodynamic and oxidation resistance properties with respect to increasing exposure to oxygen. Furthermore, the chemical-disorder can significantly change the thermodynamic and oxidation behavior of MAX phases, altering the electronic structure properties of MAX phase. This suggests a strong connection of chemical disorder with electronic and thermodynamic properties^34–37^.

In this work, we performed a detailed electronic-structure and thermodynamic analysis of Ti_2_AlC MAX phases with a A(Al/Ga)-site disorder to provide mechanisms controlling oxidation behavior. To understand the overall oxidation process, we used a high-throughput framework comprised of a machine learning model and the grand-canonical linear programming (GCLP) method and analyzed the temperature-dependence of Gibbs formation enthalpy (∆G_form_) of Ti_2_(Al_1−x_Ga_x_)C MAX phases. An in-depth thermodynamic analysis using convex hull of ternary Ti–Al–C, Ti–Ga–C, and Ti–Al–Ga–C based MAX phase was performed to provide detailed contribution arising from electronic, chemical and vibrational entropies. Our model is able to predict reaction products, phase-fractions, and chemical activities during the high-temperature reaction processes at given temperature and oxygen content. In general, the oxidation process can be attributed to the atomic interactions that arise from varying electronic states of different species that may enable or disable the formation of different kinds of oxides. Therefore, we systematically investigated the electronic-structures of series of Ga-doped Ti_2_AlC MAX phases using ab initio methods to reveal the quantum mechanical origin of change in elemental chemical activity from alloying. To prove the charge effect on stability, we also performed chemical bonding analysis using crystal orbital Hamilton population (COHP) method. We believe that our study will guide experimentalists in understanding temperature dependent oxidation reaction processes in disorder MAX phases, exemplified for Ti_2_(Al_1−x_Ga_x_)C MAX, which can be used to understand more complex MAX phases or even 2D MXenes.

## Methods

### Electronic-structure calculation

The electronic-structure calculation was performed using density-functional theory method as implemented within Vienna Ab initio Simulation Package^38–40^. The Perdew–Burke–Ernzerhof (PBE) generalized gradient approximation (GGA) functional^41^ was employed for geometrical and electronic relaxations of Ti_2_(Ga_x_Al_1−x_)C MAX with total energy and force convergence criteria of 10^–6^ eV and 0.01 eV/Å. The Brillouin zone integration in ionic and charge self-consistency were performed on 6 × 6 × 2 and 12 × 12 × 4 k-mesh using Monkhorst–Pack method^42^ with a plane-wave cutoff energy of 520 eV, where the effect of the core electrons and interaction between the nuclei and the valence was treated by the projector-augmented wave (PAW)^43,44^. Based on the work of Söderling et al.^45^ and Giese et al.^46^, we chose PBE was chosen over LDA or meta-GGA^47,48^ exchange–correlation functionals, where it was shown that PBE provides accurate bonding properties, magnetic moments, and energetics comparing LDA or meta-GGA. The 128 atom random supercell with disorder at Al site (x = 0.25, 0.50, 0.75) in Ti_2_(Ga_x_Al_1-x_)C MAX to avoid effect arising from cell size^49^.

### High-throughput machine-learning (SISSO) framework for oxidation analysis

The machine-learning-based high-throughput method was used for assessing ∆*G*_form_, phase-prediction, and chemical activity analysis of constituent elements of Ti_2_(Ga_x_Al_1−x_)C MAX phase. The SISSO (Sure Independence Screening and Sparsifying Operator) trained machine-learning model^50^ was integrated with grand-canonical linear programming (GCLP) method^51^ into a single high-throughput framework^52,53^. The SISSO model uses DFT based formation enthalpy ($\Delta {\mathbf{H}}_{\mathbf{f}\mathbf{o}\mathbf{r}\mathbf{m}})$ database to predict temperature dependent ∆*G*_form_ **[**$=\Delta {\mathbf{H}}_{\mathbf{f}\mathbf{o}\mathbf{r}\mathbf{m}}+{\Delta \mathbf{G}}^{\mathbf{S}\mathbf{I}\mathbf{S}\mathbf{S}\mathbf{O}}\left(\mathbf{T}\right)-\sum_{\mathbf{i}}{\mathbf{x}}_{\mathbf{i}}{\Delta \mathbf{G}}_{\mathbf{i}}(\mathbf{T})$**]**^54,55^, where ${\Delta \mathbf{G}}^{\mathbf{S}\mathbf{I}\mathbf{S}\mathbf{S}\mathbf{O}}\left(\mathbf{T}\right),$ ${\Delta \mathbf{G}}_{\mathbf{i}}(\mathbf{T})$, and x are vibrational (phonon) entropy, elemental energies, and the stoichiometric weight of each element in the compound, respectively. The ∆*G*_form_ also uses DFT-calculated ground state properties, including enthalpies of formation ($\Delta {\mathbf{H}}_{\mathbf{f}\mathbf{o}\mathbf{r}\mathbf{m}}$**)** and volume. The $\Delta {\mathbf{H}}_{\mathbf{f}\mathbf{o}\mathbf{r}\mathbf{m}}$ of Ti–Al/Ga–C MAX phase was calculated as ${\mathbf{E}}_{\mathbf{t}\mathbf{o}\mathbf{t}\mathbf{a}\mathbf{l}}^{\mathbf{T}\mathbf{i}2\left(\mathbf{A}\mathbf{l}-\mathbf{G}\mathbf{a}\right)\mathbf{C}}-\sum_{\mathbf{i}}{\mathbf{n}}_{\mathbf{i}}{\mathbf{E}}_{\mathbf{i}}$**,** where ${\mathbf{E}}_{\mathbf{t}\mathbf{o}\mathbf{t}\mathbf{a}\mathbf{l}}^{\mathbf{T}\mathbf{i}2\left(\mathbf{A}\mathbf{l}-\mathbf{G}\mathbf{a}\right)\mathbf{C}}$ is the total energy of the alloy, ${\mathbf{n}}_{\mathbf{i}}$ is the number of atoms and ${\mathbf{E}}_{\mathbf{i}}$ elemental energy of atom of type ‘i’. The ${\Delta \mathbf{G}}^{\mathbf{S}\mathbf{I}\mathbf{S}\mathbf{S}\mathbf{O}}\left(\mathbf{T}\right)$ is estimated from machine learning trained Bartel model^56^:

$${\mathrm{G}}^{\mathrm{SISSO}} \left(\mathrm{eV}/\mathrm{atom}\right)=\left(-2.48\cdot {10}^{-4}\cdot \mathrm{ln}\left(\mathrm{V}\right)-8.94\cdot {10}^{-5}\cdot {\mathrm{mV}}^{-1}\right)\mathrm{T}+ 0.181\cdot \mathrm{ln}\left(\mathrm{T}\right)-0.882,$$

where V is the volume calculated from the DFT and T is the temperature. The stable Ti–O, Al–O and Ga–O oxide phases were taken from NIST-JANAF database (experiments) for validation^54,57^.

### Grand canonical linear programming (GCLP)

The GCLP method was used to minimize the free energy of the mixture to identify equilibrium alloy phases:

$$\Delta {\varvec{G}}= \sum_{{\varvec{P}}}{{\varvec{f}}}_{{\varvec{P}}}\Delta {{\varvec{G}}}_{{\varvec{P}}},$$

where the $\Delta {{\varvec{G}}}_{{\varvec{P}}}$ is the free-energy of competing crystalline phases, and ${{\varvec{f}}}_{{\varvec{P}}}$ is the phase-fraction. The $\Delta {\varvec{G}}$ expression was used to minimize the free energy and predict favorable phases from Ti–Al–Ga–C + O_2_ reactions. The GCLP breaks down the alloy system into linear equations for given temperature and oxygen content. The OQMD (Open Quantum Materials Database) was used for ΔG, phase-fractions, and chemical activity prediction across the temperature range^54,55^.

## Results and discussion

### Convex hull analysis for Ti_2_AlC and Ti_2_GaC

**Figure 1:**
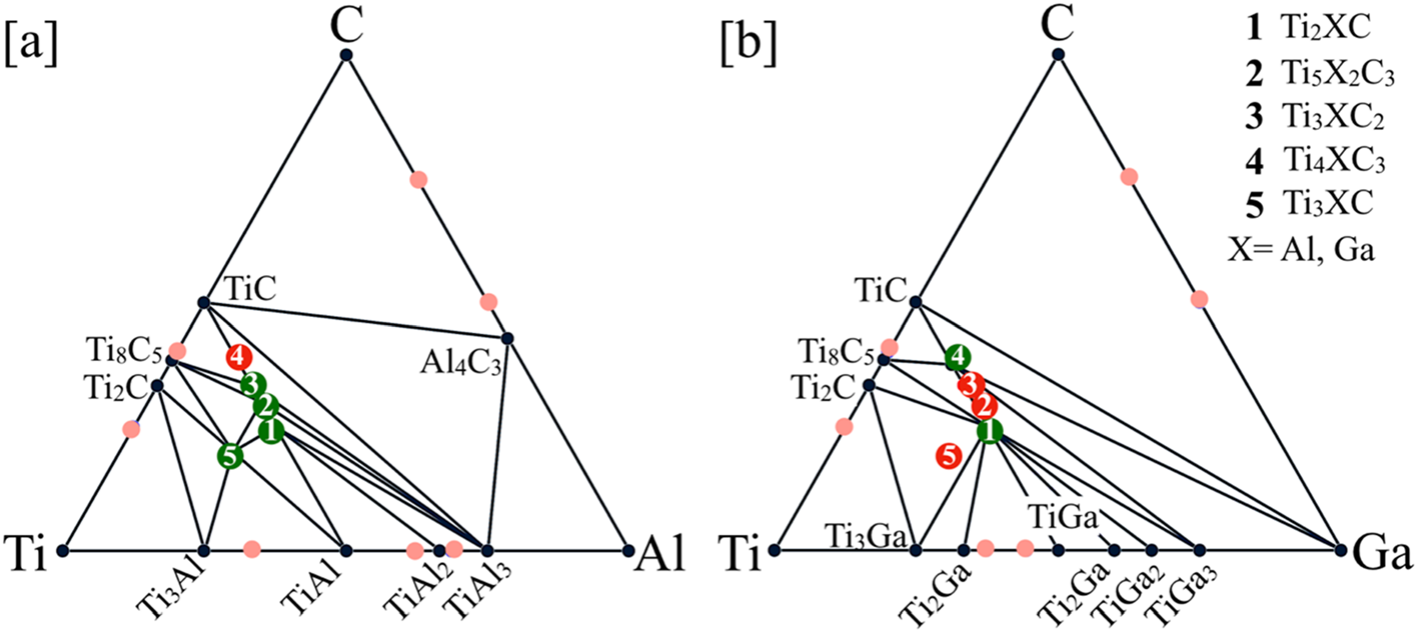
Ternary (**a**) Ti–Al–C, and (**b**) Ti–Ga–C convex hull based on experimentally known structures. The compositions marked as stable (green) and unstable (red) for given MAX phases^58^. The black and red (light) circles are stable and unstable binary/unary phases, respectively.

We constructed the ternary Ti–Al–C and Ti–Ga–C convex hull based on experimentally stable and theoretically known^56^ unstable phases in Fig. 1a,b. Notably, the coexistence of different phases occurs when a point lies on the simplex of the hull. The stable (green) and unstable (red) MAX phases including Ti_2_XC, Ti_5_X_2_C_3_, Ti_3_XC_2_, and Ti_4_XC_3_ are also marked the energies, and Δ*G*_form_ at 300 K, 1000 K, 1500 K are listed in Table 1. Other unary, binary, and ternary phases are marked. The stable phases lie on the connecting lines within convex hull formed between compounds and their elemental reference states^58,59^. The focus of our study is on the Ti_2_XC MAX phases with 2:1:1 stoichiometry, interestingly, both Ti–Al–C (#1, Fig. 1a) and Ti–Ga–C (#1, Fig. 1b) has only 2:1:1 as common stable MAX phase.

**Table 1:** The temperature dependent Gibb’s formation enthalpy (E_form_) of disorder Ti_2_(Al_1−x_Ga_x_)C MAX phases including the effect of chemical and vibrational entropy included through our machine learning framework.

x (at. Frac.)	$\Delta {{\varvec{G}}}_{{\varvec{f}}{\varvec{o}}{\varvec{r}}{\varvec{m}}}$ (eV/atom; T)			
	0 K	300 K	1000 K	1500 K
0.0	− 0.700	− 0.702	− 0.498	− 0.316
0.25	− 0.715	− 0.720	− 0.518	− 0.341
0.50	− 0.730	− 0.733	− 0.518	− 0.336
0.75	− 0.744	− 0.746	− 0.518	− 0.331
1.00	− 0.759	− 0.759	− 0.521	− 0.330

### Phase stability analysis of Ti_2_(Al_1−x_Ga_x_)C MAX phase

**Figure 2:**
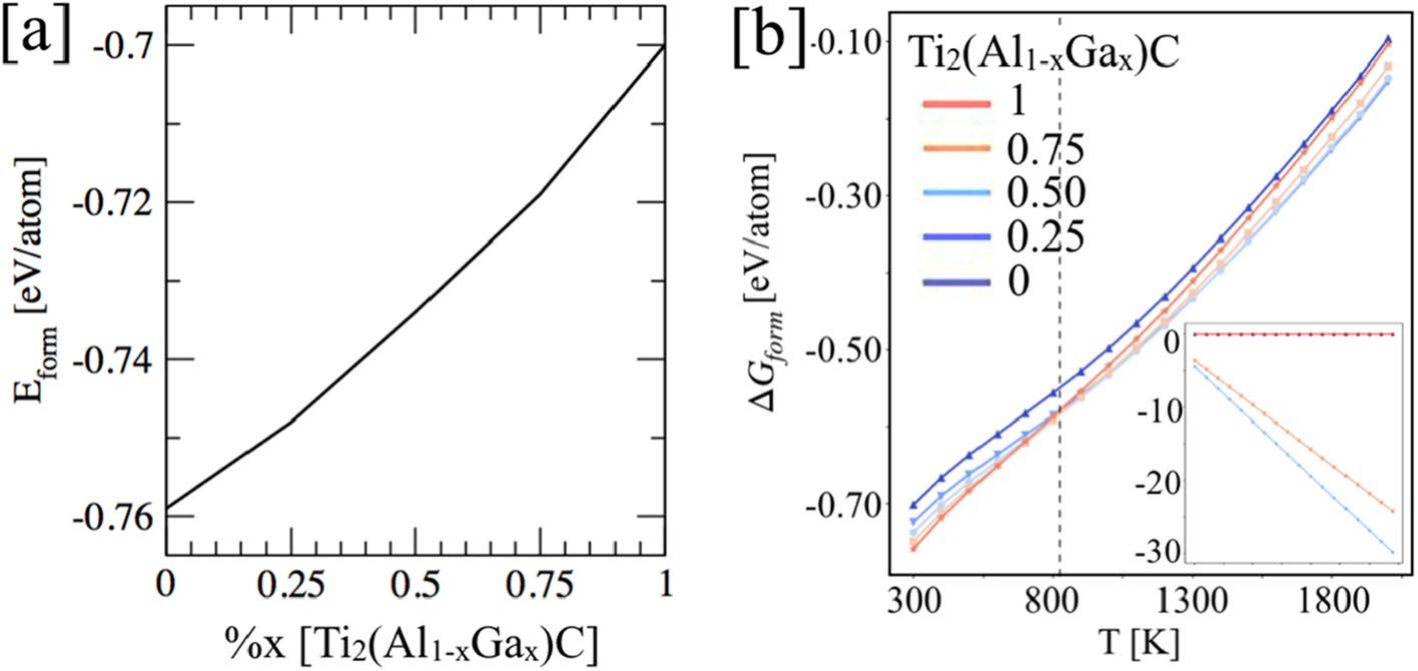
The density-functional theory predicted (**a**) formation enthalpy (0 K), and (**b**) relative phase stability of Ti_2_(Al_1−x_Ga_x_)C MAX phases with respect to constituent elements Ti, Al, Ga, and C. (**c**) The ML predicted temperature dependent $\Delta {{\varvec{G}}}_{{\varvec{f}}{\varvec{o}}{\varvec{r}}{\varvec{m}}}$ (inset shows chemical entropy contribution to the disorder phase).

The Fig. 2a shows the formation energy (E_form_) of Ti_2_(Al_1−x_Ga_x_)C calculated with the density-functional theory framework. Clearly, the increase in Ga at.% enhances the stability of Ti_2_(Al_1−x_Ga_x_)C with respect to pure Ti_2_AlC MAX phase. In Fig. 2b, we show the temperature dependent Gibbs formation enthalpy (Δ*G*_form_) of Ti_2_(Al_1−x_Ga_x_)C from 300 to 2000 K. At room temperature (RT), Ti_2_(Al_1−x_Ga_x_)C shows decrease in phase stability with increasing Al, where a crossover for Al cases at 800 K (x (Al) = 0, 0.25, 0.50, and 0.75 at.-frac.), energies are listed in Table 1. The Δ*G*_form_ shows weakly separated stability regions with respect to temperature at 800 K, i.e., below 800 K and over 800 K. The change in the Δ*G*_form_ at higher temperatures implies an interplay of temperature-dependent enthalpy and entropic contributions. This highlights the importance of temperature effect on Δ*G*_form_, which is an important quantity that allows efficient determination of the most stable equilibrium state and can thus be used to assess the resulting reaction products for a given set of reactants^52^.

**Figure 3:**
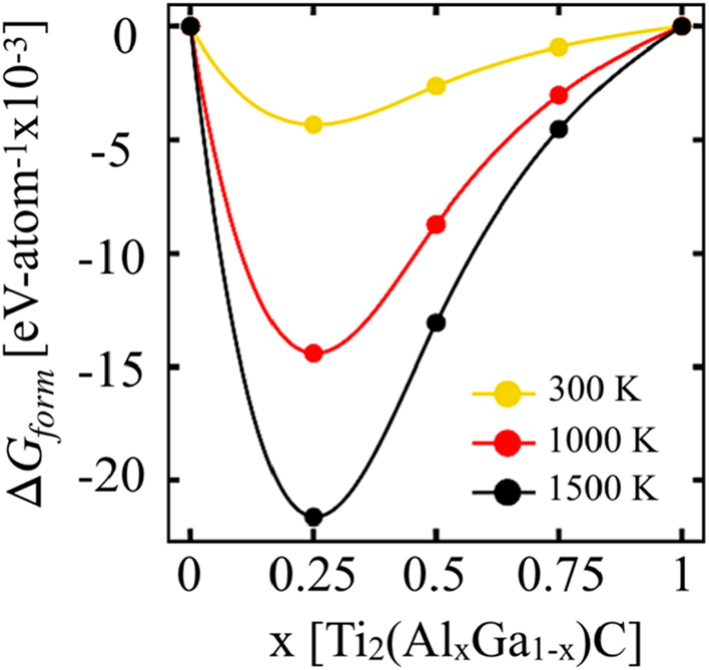
The ML predicted Gibbs formation enthalpies ($\Delta {G}_{form}$) for Ti_2_(Al_1−x_Ga_x_)C, x = 0, 1 atomic-fractions at 300 K, 1000 K, and 1500 K (see the $\Delta {G}_{form}$ in Table 1) with respect to Ti_2_GaC (x = 0) and Ti_2_AlC (x = 1).

Figure 3 shows the formation energy difference (ΔE_form_) of Ti_2_(Al_1−x_Ga_x_)C were calculated within DFT method while temperature dependence was calculated ML framework^52^. The $\Delta {{\varvec{G}}}_{{\varvec{f}}{\varvec{o}}{\varvec{r}}{\varvec{m}}}$ in Fig. 3 is plotted with respect to Ti_2_GaC (x = 0) and Ti_2_AlC (x = 1) MAX. Clearly, the increase in Ga at.% enhances the stability of Ti_2_(Al_1−x_Ga_x_)C, and the conclave slope in $\Delta {{\varvec{G}}}_{{\varvec{f}}{\varvec{o}}{\varvec{r}}{\varvec{m}}}$ further confirms the mixing of Al–Ga on A-site. However, the $\Delta {{\varvec{G}}}_{{\varvec{f}}{\varvec{o}}{\varvec{r}}{\varvec{m}}}$ with respect to end points (Ti_2_GaC and Ti_2_AlC) in Fig. 3 is very small at 300 K, i.e., increase in relative stability on Al doping does not change drastically. While increasing energy difference at higher temperatures (1000 K, 1500 K) show increasing lattice contribution as found in Table 2 (energies include electronic, chemical and vibrational entropy contributions)^52^.

**Table 2:** The formation enthalpy and entropy contributions for Ti_2_(Al_1−x_Ga_x_)C MAX phases. The electronic entropy was calculated from DFT^38–40^, the chemical entropy was estimated with relation 1/4(2 × 0 + 1 × ($\sum_{i}{c}_{i}\mathrm{ln}{c}_{i}$) + 1 × 0), and vibrational contribution to entropy was predicted from Bartel Model^56^ as implemented in our framework^52^. The volume and energy are in Å^3^-atom^−1^ and eV-atom^−1^, respectively.

x (at.-frac.)	Vol	E_form_ (0 K)	E_el_ (0 K)	E_chem_			$\mathbf{E}$_vib_		
				300 K	1000 K	1500 K	300 K	1000 K	1500 K
0	14.02	− 0.700	− 0.0039	0	0	0	− 0.0693	− 0.3638	− 0.6565
0.25	13.98	− 0.715	− 0.0039	− 0.0036	− 0.0121	− 0.0182	− 0.0757	− 0.3852	− 0.6886
0.50	13.94	− 0.730	− 0.0038	− 0.0045	− 0.0149	− 0.0224	− 0.0760	− 0.3865	− 0.6904
0.75	13.90	− 0.744	− 0.0039	− 0.0036	− 0.0121	− 0.0182	− 0.0764	− 0.3877	− 0.6923
1.00	13.87	− 0.759	− 0.0041	0	0	0	− 0.0776	− 0.3916	− 0.6982

### Model validation

**Figure 4:**
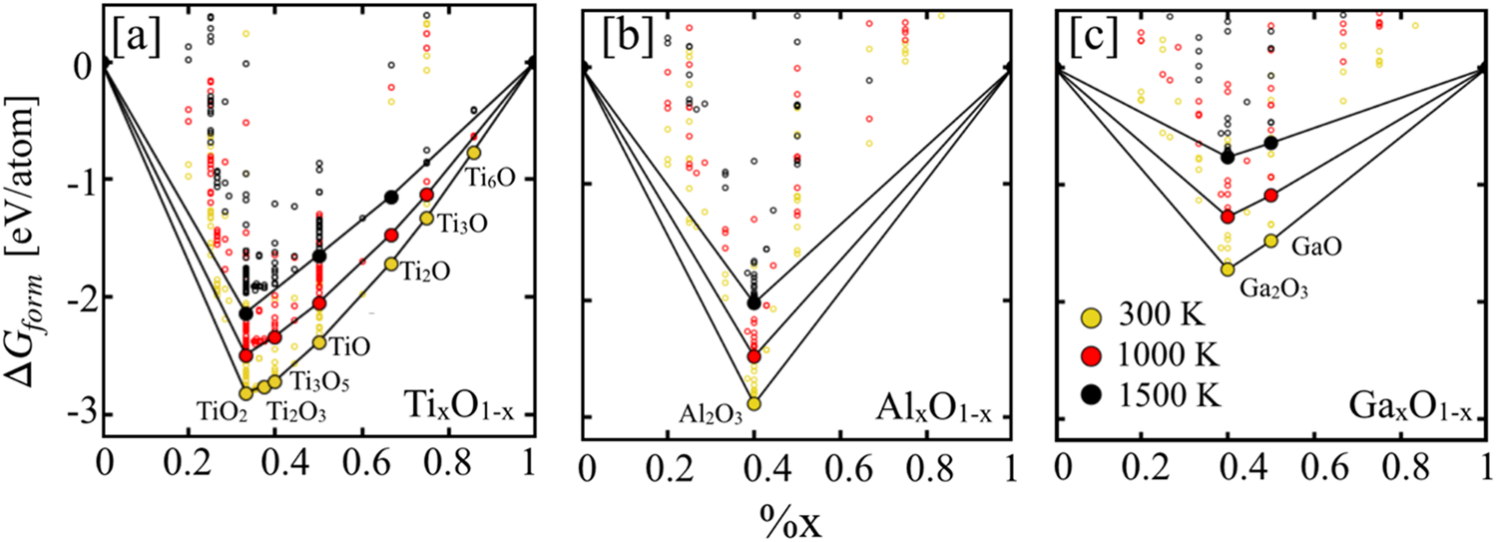
The temperature dependent convex hull of (**a**) Ti_x_O_1−x_, (**b**) Al_x_O_1−x_, and (**c**) Ga_x_O_1−x_ shows the higher stability (Gibbs formation enthalpy) of key oxide phases TiO_2_, Al_2_O_3_ and Ga_2_O_3_, which is in agreement with phases present NIST-JANAF thermochemical table^54,58^.

The model evaluation is a very important criteria for the validation of theoretical predictions or framework, we calculate composition and temperature-dependent convex hull for Ti_x_O_1−x_ Al_x_O_1−x_, and Ga_x_O_1−x_ as shown in Fig. 4a–c to validate our predictions. The convex hull in Fig. 4 is the outcome of Gibb’s enthalpy analysis at a given temperature, which uses enthalpy of formation of different phases taking part in equilibrium. The convex hull tells you that all phases on the hull compete successfully for equilibrium against other phases.

Our prediction of phase stability for Ti_x_O_1−x_, Al_x_O_1−x_, and Ga_x_O_1−x_ based oxides was found in good agreement with experimental phases in NIST-JANAF thermochemical table^54,58^. Framework^52^ predicts eight stable phases for Ti–O (TiO_2_, Ti_2_O_3_, Ti_3_O_5_, TiO_3_, TiO, Ti_2_O, Ti_3_O, and Ti_6_O) in Fig. 4a, one for Al-–O (Al_2_O_3_) in Fig. 4b, and two phases in Ga–O (Ga_2_O_3_, GaO)^58^ in Fig. 4c at 300 K. We show that Al_2_O_3_ and (GaO, Ga_2_O_3_) remains stable throughout the temperature range, while for Ti–O, two phases (Ti_2_O; Ti_6_O) disappear at 1500 K, which is in agreement with the phases reported in high-temperature thermochemical NIST-JANAF dataset^54^. Some of the phases of Ti_x_O_1−x_ that undergo a phase change at higher temperatures are ignored^56^. The TiO_2_, Al_2_O_3_ and Ga_2_O_3_ oxide phases that considered key for oxidation resistance remain most stable oxide phases, which may play a key role during oxidation process in disorder Ti_2_(Al_1−x_Ga_x_)C MAX phase. This is not straightforward conclusion as changing chemistries can have unexpected effects on phase formation and stability; therefore, it would be interesting to see how oxidizing environment impacts our final products and material survivability.

### Oxidation analysis of Ti_2_(Al_1−x_Ga_x_)C MAX phases

**Figure 5:**
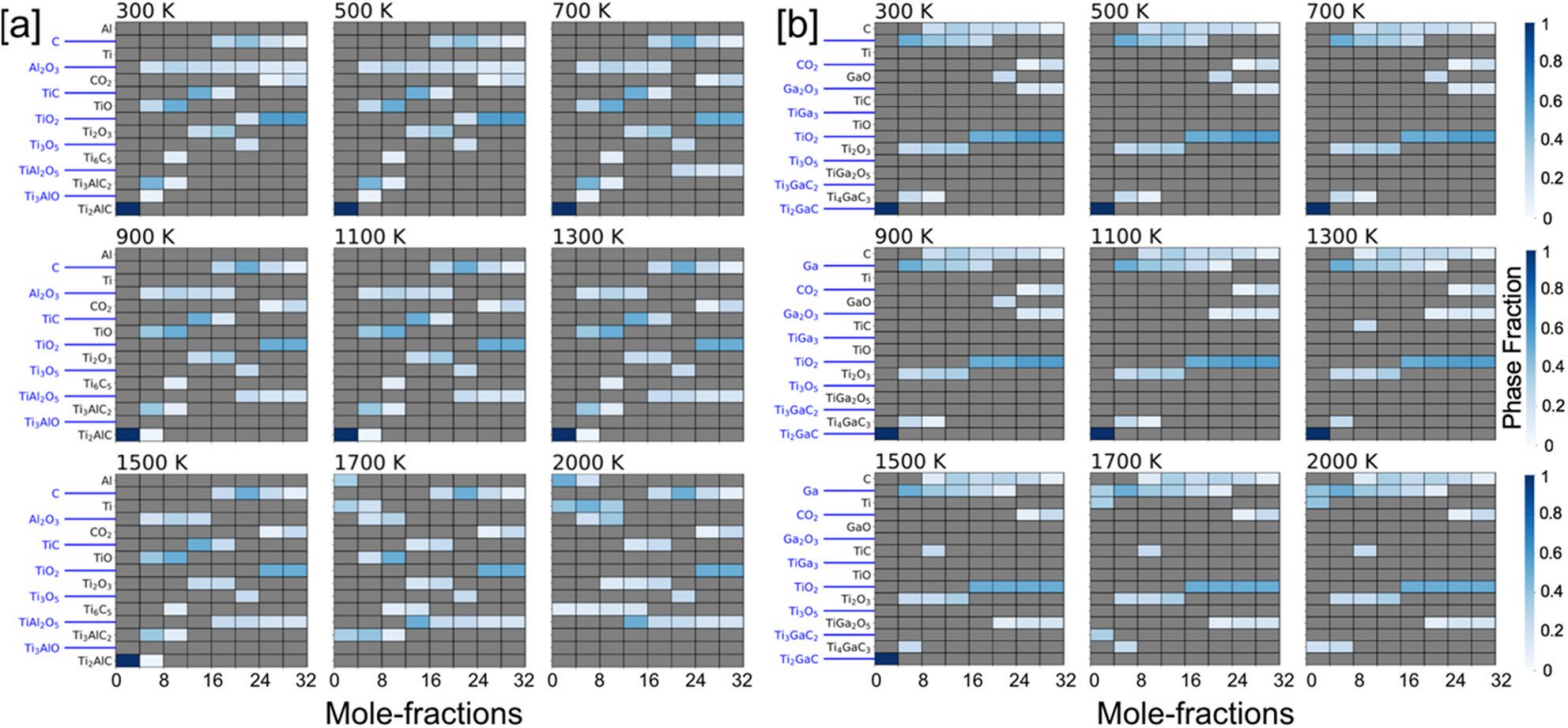
Phase fractions of (**a**) [Ti_2_AlC + O_2_] and (**b**) [Ti_2_GaC + O_2_] reaction products at different temperatures with increasing temperature from 300 to 2000 K with respect to molar percent oxygen (0–32 mol). The color gradient (shades of blue) shows molar phase fractions. Blank spot [gray (0)] suggests no phases.

(i) *x (Al) = 0.0 and 1.0* In Fig. 5a,b, we analyze the oxidation behavior of Ti_2_AlC/Ti_2_GaC and show the heat map of the molar phase-fractions of reaction products (the color bar on right represents the molar percent, or phase fraction, of each phase). In Fig. 5a, the heatmap shows the presence of Al_2_O_3_ at all temperatures 300–2000 K, whereas different Ti–O phases are observed at low (TiO), intermediate (TiO, Ti_2_O_3_, Ti_3_O_5_), and high (TiO_2_) oxygen contents. At the onset of the oxidation process Al_2_O_3_, Ti_3_AlC_2_, and TiO forms first, which is followed by the reaction of oxygen with Ti_3_AlC_2_ that gradually transforms into TiC. At high oxygen contents, the MAX phase eventually disintegrates completely into solid Al_2_O_3_ and TiO_2_, and gaseous CO_2_ phase^52^. In Fig. 5a, the heat map for Ti_2_GaC + O_2_ shows the presence of Ga_2_O_3_ up to 1300 K. On the other hand, different Ti–O phases are observed similar to Ti_2_AlC in Fig. 5a. At the onset of oxidation process and intermediate oxygen molar fractions Ga_2_O_3_ and GaO were observed. While higher order MAX phases such as Ti_3_GaC_2_, and Ti_4_GaC_3_ were found competing both at low and higher temperatures but at low oxygen content. The spinel type TiGa_2_O_3_ phase was observed at higher temperatures and high oxygen content. Both for Ti_2_AlC and Ti_2_GaC in Fig. 5a,b, the TiO forms first, which followed by the reaction of oxygen with higher order MAX phases gradually transforms to TiC. At high oxygen contents, the MAX phase eventually disintegrates completely into solid Al_2_O_3_, TiO_2_, and gaseous CO_2_ phase, which is in agreement with our oxide phase discussion in Figs. 3 and 4.

To understand the reaction mechanism arising changing chemical potential on varying molar oxygen percent, which represents the exposure time of the alloy to static air in experimental conditions. The reaction products of the chemical process during the selective oxidation of Al/Ga or Ti in Ti_2_AlC/Ti_2_GaC can be written as:

1 $${\text{4Ti}}_{2} {\text{AlC}} + 3y \cdot {\text{O}}_{2} \to {\text{4Ti}}_{2} {\text{Al}}_{{1 - {\text{x}}}} {\text{C}} + 2y \cdot {\text{Al}}_{2} {\text{O}}_{3} ,$$

and

2 $${\text{4Ti}}_{2} {\text{GaC}} + 3y \cdot {\text{O}}_{2} \to {\text{4Ti}}_{2} {\text{Ga}}_{{1 - {\text{x}}}} {\text{C}} + 2y \cdot {\text{Ga}}_{2} {\text{O}}_{3} ,$$

3 $${\text{Ti}}_{2} {\text{AlC}} + 2y \cdot {\text{O}}_{2} \to {\text{2Ti}}_{{2 - {\text{2x}}}} {\text{AlC}} + 2y \cdot {\text{TiO}}_{2} ,$$

and

4 $${\text{Ti}}_{2} {\text{GaC}} + 2y \cdot {\text{O}}_{2} \to {\text{2Ti}}_{{2 - {\text{2x}}}} {\text{GaC}} + 2y \cdot {\text{TiO}}_{2} .$$

Considering longer exposure time to static air, C diffuses through TiO_2_ and oxidizes into CO_2_^–^

5 $${\text{Ti}}_{{2}} {\text{AlC}} + {\text{O}}_{{2}} \to {\text{Al}}_{{2}} {\text{O}}_{{3}} + {\text{TiO}}_{{2}} + {\text{CO}}_{{2}} ,$$

and

6 $${\text{Ti}}_{{2}} {\text{GaC}} + {\text{O}}_{{2}} \to {\text{Ga}}_{{2}} {\text{O}}_{{3}} + {\text{TiO}}_{{2}} + {\text{CO}}_{{2}} ,$$

i.e., C from Ti-C diffuses through the mixed Ti-oxides layer and oxidize. The diffusion of Ti to the surface and O into the MAX phase or oxidation product during the oxidation process works as the rate-limiting condition.

### Chemical activity of constituent elements of the [Ti_2_AlC/Ti_2_*GaC* + O_2_]

**Figure 6:**
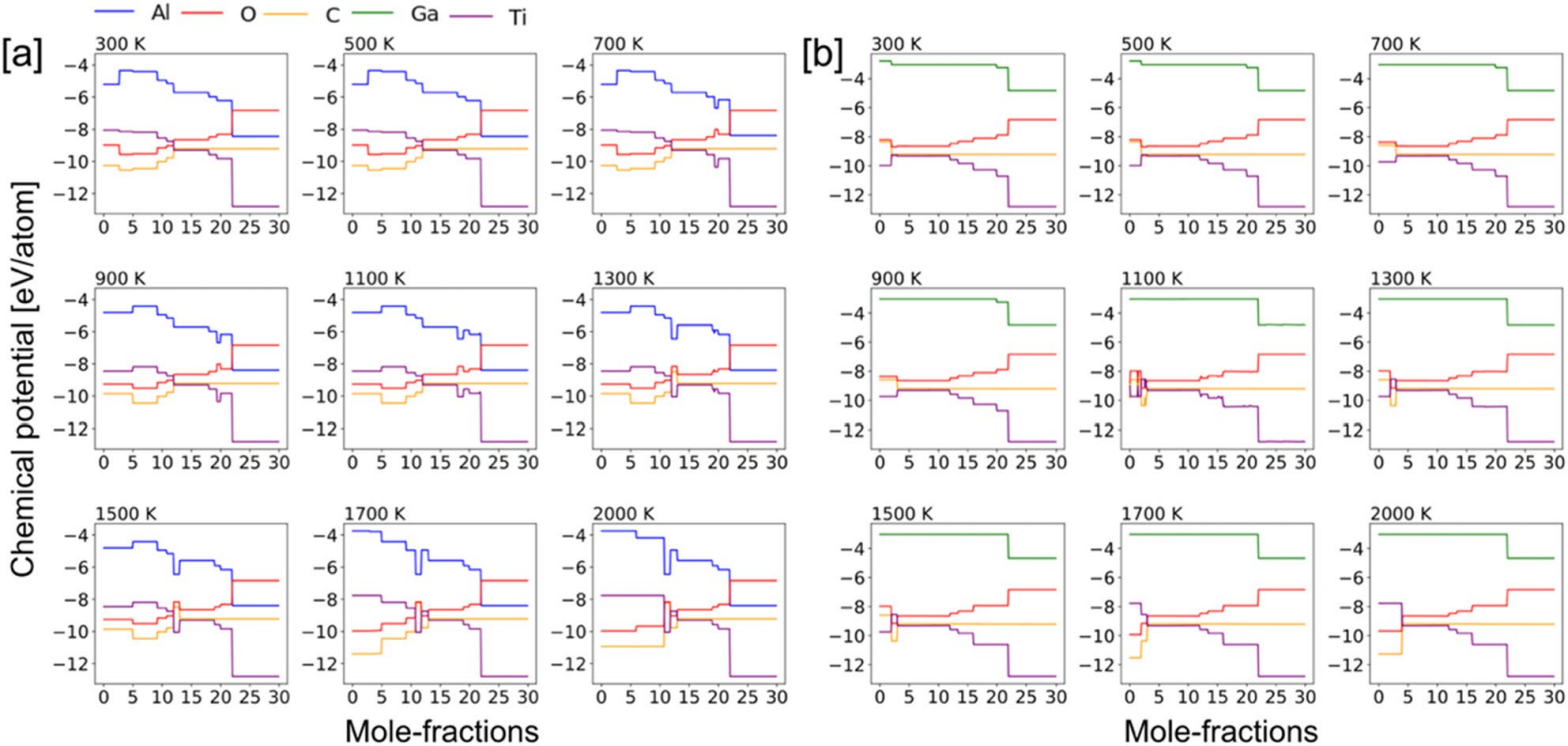
Oxidation reaction chain showing change in chemical potential of (**a**) [Ti_2_AlC + O_2_] and (**b**) [Ti_2_GaC + O_2_] as a function of changing molar percent oxygen from 300 to 1200 K. On oxidation, the partial chemical potentials of Ti/Al/Ga/C reduce while the chemical potential of invading O_2_ increases.

The chemical activity of constituent elements of [Ti_2_AlC + O_2_] and [Ti_2_GaC + O_2_] during the oxidation process are shown in Fig. 6a,b. The (partial) chemical potential of (Ti, Al/Ga, C, O) is calculated for an open system using unknown molar concentration of reaction products by mixing of grand-canonical ∆*G*_form_ from 300 to 2000 K. The reaction chain is associated with the reductions in the partial chemical potentials of Ti and Al, but an increase in the chemical potential of O with increasing oxygen content while C remains almost unchanged except at high oxygen content and high temperature. The higher Ti/Al activity at the early oxidation stage is directly related to their partial chemical potentials. Two chemical potentials zones in Fig. 6a,b with increasing oxygen content are identified—(a) slowly varying (0–20 mol oxygen); and (b) sharp changing (> 20 mol oxygen). The sharp change in chemical potential occurs in the region > 10 mol oxygen as Ti_3_AlC_2_, Ti_3_GaC_2_, Ti_4_GaC_3_, Ti_3_AlC_2_, TiC completely disintegrates by then, moreover, C oxidizes to form gaseous CO_2_. The occurrence of C and CO_2_ at higher temperature suggests loss of carbon. The predicted trend in chemical potential suggests increased oxygen activity at higher oxygen content. It is obvious from Fig. 6a,b that Al activity is higher than Ga in MAX phase. This also could be correlated to Ellingham diagrams, where Al_2_O_3_ has much lower partial pressure compared to Ga_2_O_3_.

### Elemental chemical potentials

**Figure 7:**
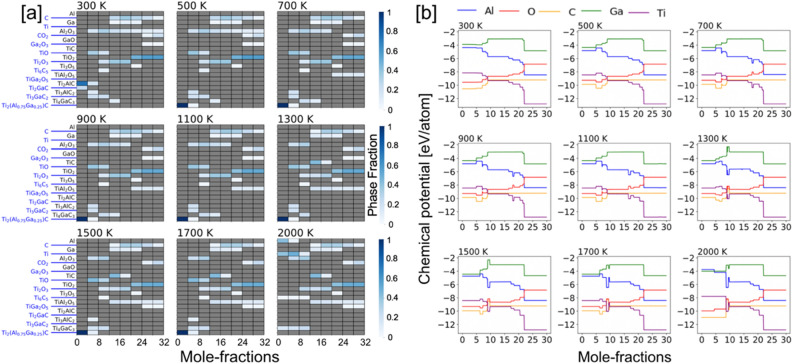
(**a**) Phase fractions and (**b**) chemical potential of [Ti_2_(Al_0.75_Ga_0.25_)C + O_2_] with increasing temperature from 300 to 2000 K and molar percent oxygen (0–32 mol). The color gradient (shades of blue) shows molar phase fractions. Blank spot [gray (0)] suggests no phases. On oxidation, the partial chemical potentials of Ti/Al/Ga/C reduce while the chemical potential of invading O_2_ increases.

**Figure 8:**
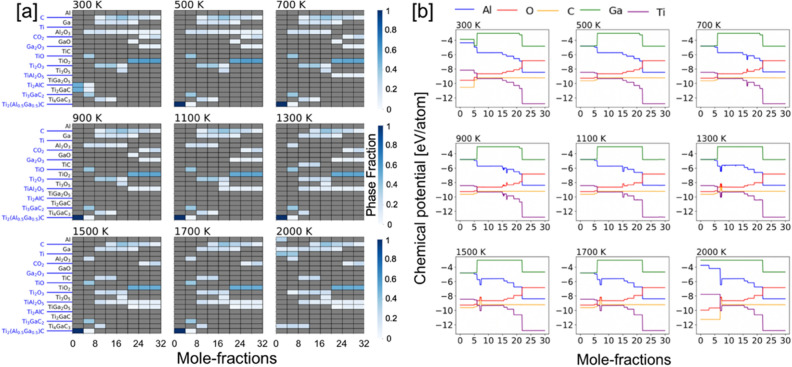
(**a**) Phase fractions and (**b**) chemical potential of [Ti_2_(Al_0.50_Ga_0.50_)C + O_2_] with increasing temperature from 300 to 2000 K and molar percent oxygen (0–32 mol). The color gradient (shades of blue) shows molar phase fractions. Blank spot [gray (0)] suggests no phases. Upon oxidation, the partial chemical potentials of Ti/Al/Ga/C reduce while the chemical potential of invading O_2_ increases.

**Figure 9:**
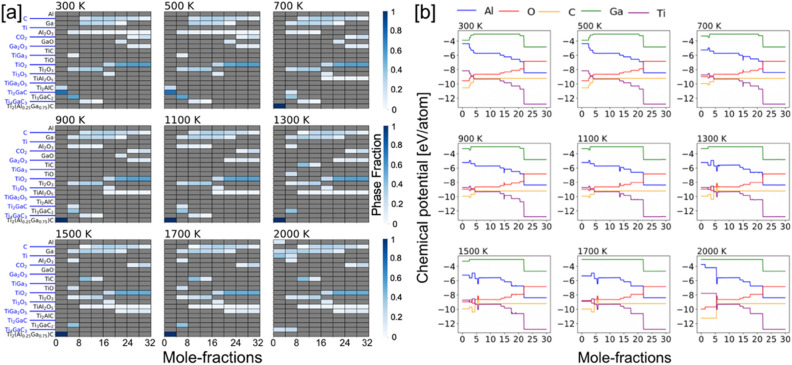
(**a**) Phase fractions and (**b**) chemical potential of [Ti_2_(Al_0.25_Ga_0.75_)C + O_2_] with increasing temperature from 300 to 2000 K and molar percent oxygen (0–32 mol). The color gradient (shades of blue) shows molar phase fractions. Blank spot [gray (0)] suggests no phases. On oxidation, the partial chemical potentials of Ti/Al/Ga/C reduce while the chemical potential of invading O_2_ increases.

Our framework treats all elemental reservoirs as an ideal gas model. The resulting phases at a particular temperature are determined by those that result in a minimal energy of the system. At any temperature the amount of oxygen is incrementally increased over a range of values, i.e., a variable oxygen reservoir. As more oxygen becomes available, greater oxygen dense phases are predicted to form Ti_2_O over TiO_2_. From a particular profile, we can solve for the individual chemical potentials using the GCLP model via relation μ_i_ = $\partial U/\partial N$, where μ_i_ is the chemical potential of `i’ element. The chemical potential of an oxygen gas molecule changes with temperature and partial pressure, however, we considered partial pressure of 1 atm in all our calculations assuming experimental conditions with normal pressure. The accepted energy of oxygen gas phase was calculated from DFT at (0 K), which is − 9.67 eV per atom, and listed in the OQMD dataset in the same manner as other unary components. The nominal change in oxygen ratio during oxidation reaction at each temperature changes the chemical potential when we solve grand canonical linear programming (GCLP) method. The temperature dependence of the chemical potential of oxygen is considered from the thermo-chemical tables^54^.

(ii) *x (Al) = 0.75, 0.50, 0.25* We show the phase-factions with increasing Ga at.% disorder at Al site of Ti_2_AlC in Figs. 7, 8 and 9a, while respective chemical potentials are shown in Figs. 7, 8 and 9b. The heatmap of the molar phase-fractions of Ti_2_(Al_0.75_Ga_0.25_)C + O_2_ reaction product in Fig. 7a shows the presence of Al_2_O_3_ at all oxygen mole fractions and for 300–2000 K. On Ga alloying, a new Ti–O phase, Ti_6_O_11_, was observed at higher temperature and high oxygen. The oxidation reactions at low intermediate, and high oxygen exposure shows TiO, (TiO, Ti_2_O_3_, Ti_3_O_5_), and TiO_2_ phases, respectively. We also predict the formation of a new complex oxide Ti–Ga–O phase, i.e., TiGa_4_O_8_. At the onset of oxidation process at higher temperature (> 700 K), the A-site disordered Ti_2_(Al_0.75_Ga_0.25_)C MAX phase shows improved stability, which slowly transforms into Al_2_O_3_, Ti_2_AlC, Ti_2_GaC and TiO with increasing oxygen. Both Ti_3_AlC_2_ and Ti_4_GaC_3_ gradually transforms to TiC at higher temperature, and the MAX phase eventually disintegrates completely into solid Al_2_O_3_ and TiO_2_, and gaseous CO_2_ phase at high oxygen contents. We found a transient Ga based oxide Ga_2_O_3_ up to 1300 K at medium oxygen molar content and disappears at higher temperatures. The observations discussed were found true for all x(Ga) cases, i.e., x = 0.75, 0.50, 0.25 at.-frac. in Figs. 7, 8 and 9a.

We rewrote the Eqs. (1–3) which now include Ga Al-site, where we varied the molar oxygen percent and tracked the change chemical activity of elements as shown in Figs. 7, 8 and 9b, which represents the exposure time of the alloy to static air in experimental conditions. The reaction products of the chemical process during the selective oxidation of Al/Ga or Ti in Ti_2_(Al_0.75_Ga_0.25_)C can be written as:

7 $${\text{4Ti}}_{2} \left( {{\text{Al}}_{{0.75}} {\text{Ga}}_{{0.25}} } \right){\text{C}} + 4y \cdot {\text{O}}_{2} \to {\text{4Ti}}_{2} \left( {{\text{Al}}_{{0.75}} {\text{Ga}}_{{0.}} } \right)_{{1 - {\text{x}}}} {\text{C}} + 2y \cdot {\text{Al}}_{2} {\text{O}}_{3} + 2y \cdot {\text{Ga}}_{2} {\text{O}}_{3} ,$$

and

8 $${\text{Ti}}_{{2}} \left( {{\text{Al}}_{{0.{75}}} {\text{Ga}}_{{0.{25}}} } \right){\text{C}} + {2}y \cdot {\text{O}}_{{2}} \to {\text{2Ti}}_{{{2} - {\text{2x}}}} \left( {{\text{Al}}_{{0.{75}}} {\text{Ga}}_{{0.{25}}} } \right){\text{C}} + {2}y \cdot {\text{TiO}}_{{2}} + {\text{ TiAl}}_{{2}} {\text{O}}_{{5}} + {\text{TiGa}}_{{2}} {\text{O}}_{{5}} .$$

Considering longer exposure time to static air, C diffuses through TiO_2_ and oxidizes into CO_2_ while we also observe that the Ga based intermetallic phase shows very high stability.

9 $${\text{Ti}}_{{2}} \left( {{\text{Al}}_{{0.{75}}} {\text{Ga}}_{{0.{25}}} } \right){\text{C}} + {\text{O}}_{{2}} \to {\text{Al}}_{{2}} {\text{O}}_{{3}} + {\text{TiO}}_{{2}} + {\text{ GaO}} + {\text{Ga}}_{{2}} {\text{O}}_{{3}} + {\text{CO}}_{{2}} .$$

### Chemical activity of Ti/Al/Ga/C in Ti_2_(Al_1−x_Ga_x_)C

The chemical activity of the constituent elements in [Ti_2_(Al_1−x_Ga_x_)C + O_2_] during the oxidation process are shown in Figs. 7, 8 and 9b. The (partial) chemical potential of (Ti, Al/Ga, C, O) is shown from 300 to 2000 K and is calculated by the mixing of ∆*G*_form_ using GCLP. Similar to Ti_2_AlC, the reaction chain associated with the reductions in the partial chemical potentials of Ti and Al is shown. The chemical potential of Ga slightly increases then decreases, possibly due to the appearance of metastable oxide phase GaO, which disappears at higher temperature and high oxygen content. The increase in the O chemical potential with increasing oxygen content while C remains almost unchanged or weakly changed except at high oxygen content and high temperature. The higher Ti/Al activity compared to Ga at the early oxidation stage is directly related to their partial elemental chemical potentials. Two chemical potentials zones in Figs. 7, 8 and 9b with increasing oxygen content are identified—(a) slowly varying (0–10 mol oxygen); and (b) sharp changing (> 10 mol oxygen). The sharp change in chemical potential occurs in the region > 10 mol oxygen as Ti_2_AlC_2_/Ti_3_AlC_2_/TiC completely disintegrates by then, moreover, C oxidizes to form gaseous CO_2_. The occurrence of C and CO_2_ at higher temperature suggests loss of carbon. The predicted trend in chemical potential suggests increased oxygen activity at higher oxygen content. Similarly, for Ga doped cases, we observed that chemical activity of Al increased in the intermediate and higher exposure to oxygen at all temperatures compared to pure Ti_2_AlC in Fig. 6. This suggests that Ga can be used as a catalyst in Al based MAX phases to control the elemental chemical activity.

## Discussion

The Al_2_O_3_ was seen at all temperatures and all oxygen concentrations due to the high chemical activity of Al, see Figs. 5, 7, 8 and 9a, as well as the very exothermic nature of the Al_2_O_3_ phase itself. The weak metallic bonding between Ti–Al (2.84 Å) compared to Ti-Ga (2.79 Å) also contribute to the increased Al diffusivity^60^, which results into later appearance of Ga-based phases at low temperature and low oxygen content. The formation of Al_2_O_3_ results into Al/Ga depletion in Ti_2_(Al_1−x_Ga_x_)C at the early-stage oxidation. This in turn leads to the decomposition of the MAX phases into TiO, Al_2_O_3_, and Ti_2_AlC/Ti_2_GaC, Ti_3_AlC_2_ and Ti_4_GaC_3_ at low oxygen concentration. This indicates that Ti and Al/Ga are the first oxidizing elements when Ti_2_(Al_1−x_Ga_x_)C in Figs. 7, 8 and 9 was exposed to ambient air at elevated temperatures. On further increasing the oxygen concentrations, the Al_2_O_3_ oxide scale remains stable compared to other oxides as partial pressure to form Al_2_O_3_ is much lower than of TiO_2_^61^ or Ga based phases^62^. The weaker binding of Al with C or Ti^63,64^ in Ti_2_(Al_1−x_Ga_x_)C compared to Ga and better Al diffusion^60^ eases the Al_2_O_3_ growth. The better thermodynamic stability of Al_2_O_3_ compared to other phases during oxidation of Ti–Al–Ga–C also helps in stabilizing Al_2_O_3_ at elevated temperatures. The C and CO_2_ appear as the reaction products at higher temperature, which suggests C loss and the evaporation of CO_2_ from the oxide scale^62^. The reaction product of in Ti_2_(Al_1−x_Ga_x_)C oxidation reaction correctly reproduces experimentally observed phase-fractions. We also found that Al_2_O_3_ forms at all temperature and all oxygen contents but Al_2_O_3_ stops forming at very high-T and high-O_2_ due to higher stability of spinel phase oxides. The appearance of a more stable spinel oxide phase hampers the Al_2_O_3_ formation, which is the reason Al_2_O_3 _is not observed at high-T. For clarity, however, the Al_2_O_3_ formed at the early oxidation stage will remain in the system and work as a protective layer. Notably, the spinal phase becomes more favorable both due to its favorable thermodynamics and requirement of low Al content.

### Electronic-structure of Ti_2_(Al_1−x_Ga_x_)CMAX

**Figure 10:**
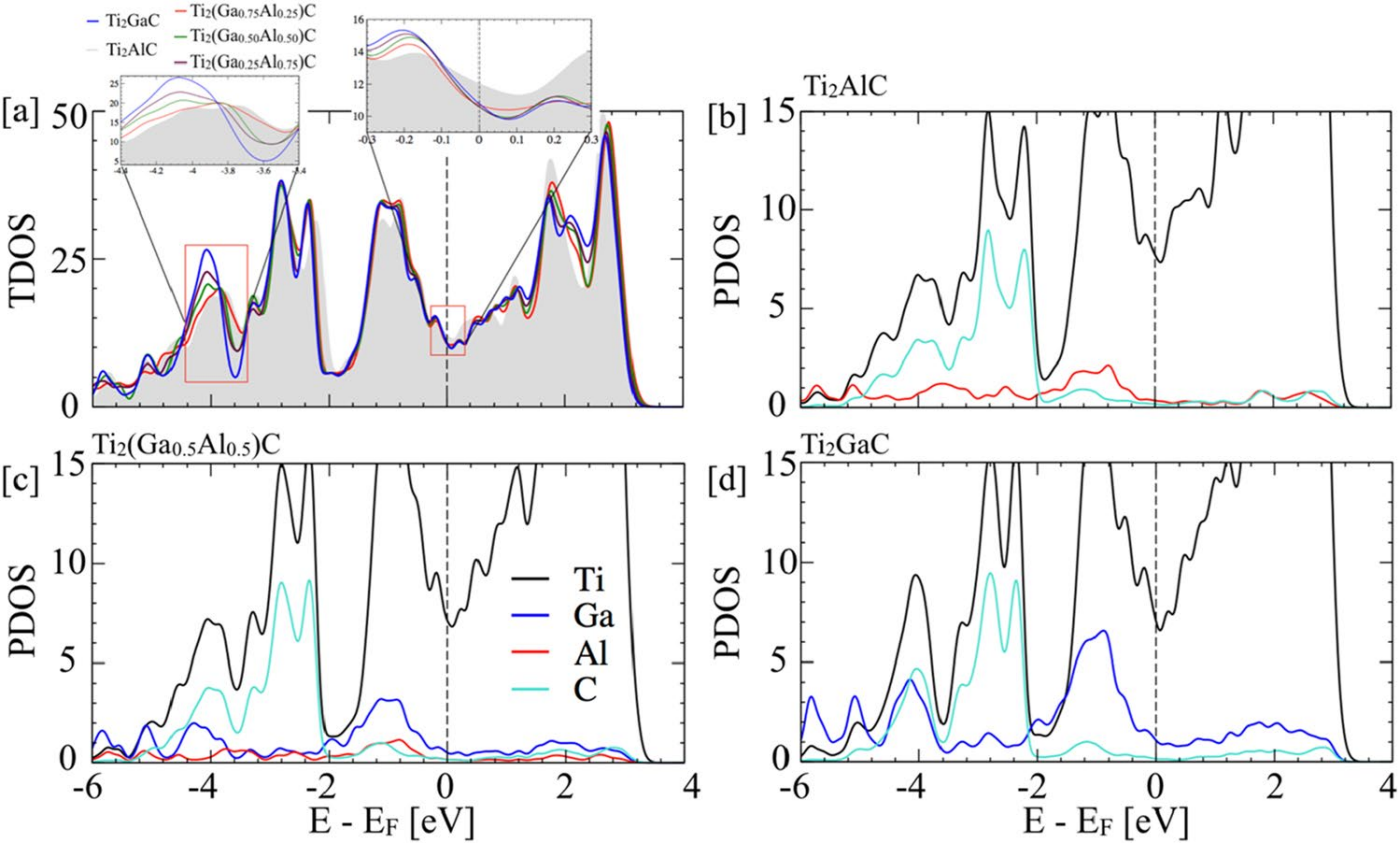
(**a**) The total density of states (DOS) with A-site disorder by Ga in Ti_2_AlC MAX. (**c**,**d**) The partial DOS of Ti_2_(Al_1−x_Ga_x_)C for x = 0, 0.50, and 1. The crystal orbital Hamilton population (COHP) analysis for Ti_2_AlC and Ti_2_GaC is provided later in bond analysis section.

**Figure 11:**
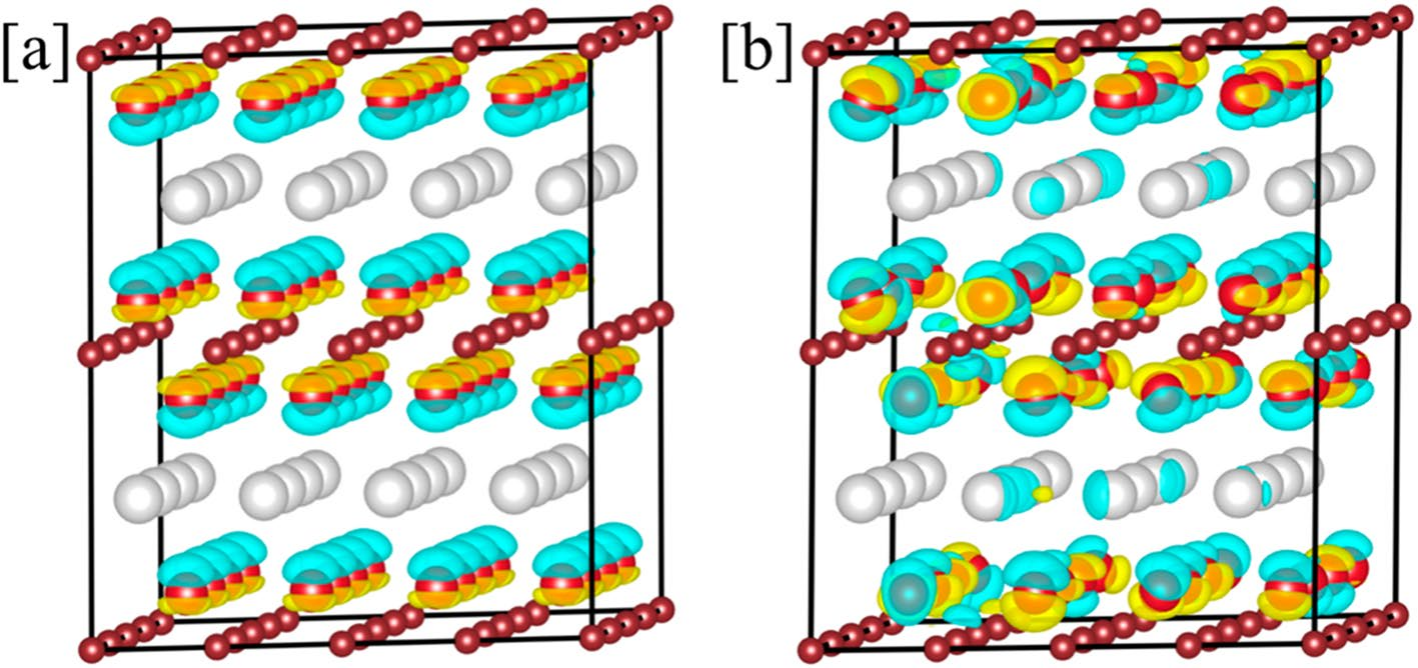
The charge density difference (Δρ) for (**a**) ρ (x = 1.0)–ρ (x = 0), and (**b**) ρ (x = 1.0)–ρ (x = 0.50) in Ti_2_(Al_1−x_Ga_x_)C MAX with respect to Ti_2_AlC, x is represented in atomic fractions.

As discussed in the oxidation section, the chemical activity of Al has increased as the Ga reduces the interaction with M–A layer. The question arises that what changes in the electronic structure correlates with the oxidation behavior in the MAX phase. The detailed electronic nature of Ti_2_(Al_1−x_Ga_x_)C was analyzed using electronic density of states (DOS) and charge-density difference in Figs. 10 and 11, respectively.

The DOS is an effective tool to reveal the hybridization among the different electronic states. In Fig. 10a–d, we plot total and partial DOS to understand the effect of Ga alloying, where the Fermi level (E_Fermi_) is set at zero. Our goal is to understand to role of electronic structure on oxidation behavior of Ti_2_(Al_1-x_Ga_x_)C MAX. The Ga doping in Fig. 10a shows strong change Ti-3*d* and Ga-4*p* states near − 4 eV and − 1 eV as shown in two highlighted zones. Clearly, the DOS in Ga doped cases is significantly reduced compared to parent phase, i.e., Ti_2_AlC MAX. The prime contribution at the E_F_ in the DOS comes from the Ti-3*d* ([Ar]3*d*^2^4*s*^2^) states as shown in Fig. 10b–d, while Ga-4*p* ([Ar]4*s*^2^4*p*^1^) and Al-3*p* ([Ne]3*s*^2^3*p*^1^) also contribute but lesser in magnitude compared to the Ti-3*d*. The valence states could be divided into three energy range, i.e., (i) − 12.0 eV to − 9.0 eV, (ii) − 9.0 eV to − 2.0 eV, and (iii) − 2.0 to 0.0. The DOS at the lower energies in the region (i) show peaks at nearly − 10 eV is mainly comprised of Ti-3*d* and C-2* s* below the *E*_F_ (not shown in the Fig. 10 as they are not chemically activity), which gives rise to the formation of stronger Ti–C bonds. While energy states for Ga doped cases in the region (ii) comprised of Ti-3*d*, Al-3*p*, and Ga-4*p* in Fig. 10b–d. Notably, in the region (iii), we found an increased Ga-4*p* states near the E_F_, which shows strong overlap with Ti-3*d* bands.

The DOS in both parent and Ga-doped phases are mainly contributed by the Ti-3*d*^2^, C-2*p*^*1*^, and Al-3*p*^1^ states. The Ti-3*d* and Ga-4*p*/Al-3*p* hybridized strongly that lead the peaks in the low energy region of both total and partial DOS. The energy region of 0 to − 6 eV is dominated by Ti-3*d* which is also hybridized with the Ga-4*p*/Al-2*p*. The peaks in the DOS in Fig. 10 are the consequence of the hybridization between different orbitals that defines the energy of the hybridized states. The peak positions show weak shift both in the valence and the conduction bands, which is expected to have no major effects on electronic structure. Meanwhile, the partial DOS analysis in Fig. 10b–d, shows changes in hybridization between Ti and C when Al is doped with Ga, while the MAX phase remains thermodynamically stable in the hexagonal phase. This change in energy of bonding states due to Ga-doping reflects the change in the Ti–C hybridization strength, consequently, the bonding strength between unlike atoms. The finite DOS at the *E*_F_ indicates that doped Ga atoms could not change the metallic character. At the top of the valence band, an interaction between Ti-3*d* and C-2*p* peaks in the energy range of − 2.0 eV and − 5 eV that leads to strong hybridization, and results into a strong directional bonding.

A peak or a valley like structure in DOS at E_F_ signifies the presence of a pseudo gap, which is a signature of both chemical and structural stability^59,65–67^. The presence of pseudo-gap region and alloys stability has its origin in several active electronic mechanisms including charge transfer and change in hybridization that pulls down the electronic DOS from E_F_ either below the Fermi level or above it^67,68^. In Fig. 10, we found that the decreases electronic density of states at E_F_ originates from change in mixing of the Ti-3*d* states with Al-3*p* due to Ga alloying, which shows increase in Ga-4*p* states near − 1 eV with increasing at.% Ga. This correlates well with the stability analysis in Figs. 1 and 2 (see Table 1). The A-site alloying weakens the bonding in Ti–Al–Ga–C MAX. This suggests that Ga can be tuned to manipulate chemical activity of Al based MAX.

The charge density analysis:

The direction of intra-layer (Ti–C/Al–Ga) and inter-layer (Al/Ga–C) charge transfer is also an important feature of MAX phase stability. In Fig. 11a,b, we plot the charge density difference ($\Delta \rho ={\rho }_{Ti2(Al1-Gax)C}$ − ${\rho }_{Ti2AlC}$) for Ti_2_(Al_1−_Ga_x_)C for x = 1.0, and 0.50, where blue color represents the charge from the Ti_2_AlC MAX while the yellow color is charge readjustment. Our charge plot in Fig. 11 shows no effective change in charge at C and Ti loses the charge. This charge asymmetry at site A-site was caused by Ga alloying in the Ti_2_AlC MAX, therefore, a varying strength of Al/Ga metallic bonds are expected. The effective charge transfer between Ga to Al shows the mechanism controlling the bonding behavior in MAX phase, which also weakens the bonding Al interaction with the Ti–C basal plane. This gain in charge density in Ti_2_GaC originates from decrease of 1.4% volume with respect to Ti_2_AlC, which is possibly one of the reasons why Al chemical activity has increased with Ga doping.

### Chemical bonding analysis

**Figure 12:**
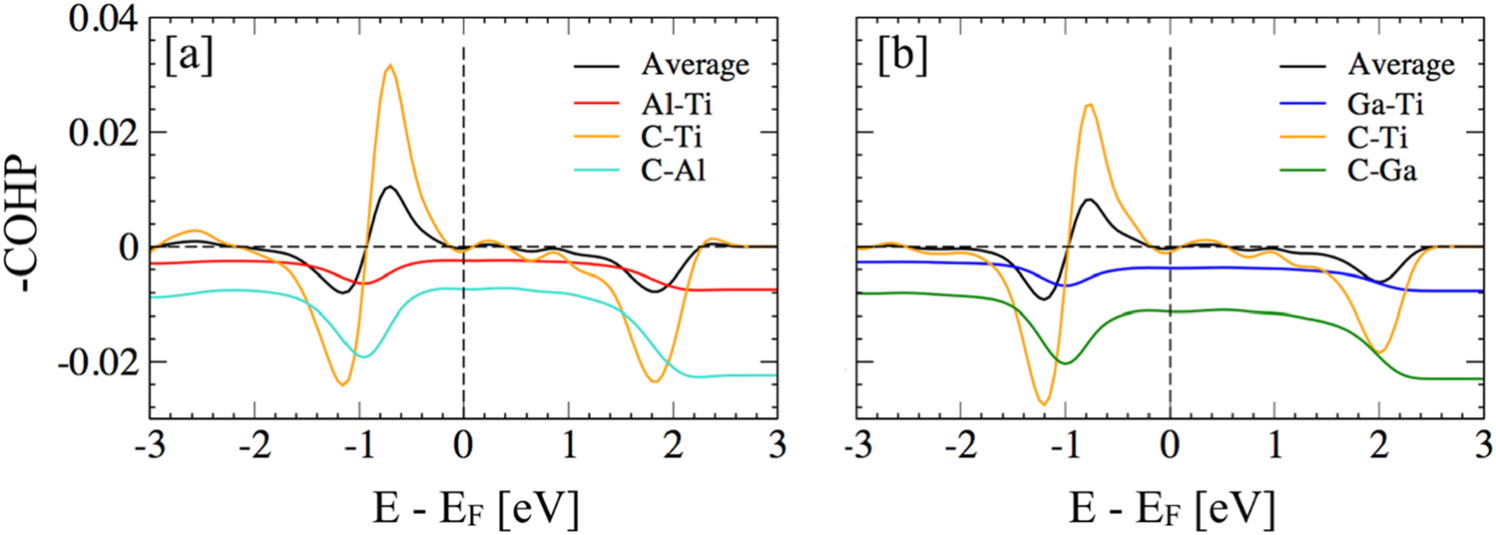
The crystal orbital Hamilton population (COHP) analysis was done for three short Al/Ga–Ti, C–Ti, C–Al/Ga bonds in Ti_2_AlC and Ti_2_GaC MAX. The energies are shown relative to the Fermi level.

We performed crystal orbital Hamilton population (COHP) analysis to elucidate the bonding behavior^69–73^. The COHP partitions the band structure energy into bonding, nonbonding and antibonding energy of atomic pair contribution in a specified energy range. In Fig. 12a,b, we plot-pCOHP as a function of energy for Ti_2_(Al_1−x_Ga_x_)C, x = 0,1. Positive values of –pCOHP describe bonding energy regions whereas negative values describe antibonding energy regions. The COHP shows slightly different picture of bonding for Ti_2_GaC MAX in Fig. 12b, where Ga shows weak reduction of C-Ti bonding strength while weak increase in anti-bonding states. This indicates towards increased charge activity due to Ga as shown by charge density difference plot in Fig. 10. Notably, the antibonding states for the C–Ti appear near − 1.2 eV below the Fermi level both for Ti_2_AlC (− 1.17 eV in Fig. 12a) and Ti_2_GaC (− 1.2 eV in Fig. 12b). Both bonding and antibonding states in Ti_2_GaC show a shift below Fermi level due to charge filling, which again corroborate with our idea of increase charge activity. This is also in agreement with the fact that the Ga doping leads to lower volume and lower enthalpy as shown in Tables 1 and 2. Expectedly, Ga/Al–Ti or Ga/Al–C show antibonding (negative COHP) in the whole energy range as Al/Ga and Ti–C are in two different basal planes in MAX phase.

## Conclusion

We systematically investigated the thermodynamic stability and oxidation behavior of Ti_2_AlC MAX phases with Ga alloying using high-throughput machine-learning framework combined with density-functional theory. The A-site (at Al) disordering of Ti_2_AlC with Ga shows significant change in the chemical activity of Al with increasing temperature and exposure to static oxygen. Our thermodynamic analysis shows changes in the Δ*G*_form_ at higher temperatures, which implies an interplay of temperature-dependent enthalpy and entropic contributions in oxidation behavior of Ga doped Ti_2_AlC MAX phase. Therefore, we have included electronic, chemical, and vibrational entropy contribution with vibrational contribution is the largest and has significant impact on high-temperature stability of MAX phases. Our convex hull analysis of key Ti–O, Al–O, and Ga–O oxides provides validation of our framework that provides accurate phase predictions, which is critical in oxidation analysis. We also show that the increased Al activity during oxidation in Ga doped MAX phases may improve the oxide layer (Al_2_O_3_) formation, which will serve as protection against oxygen diffusion by delaying the formation Ti–O (e.g., TiO_2_) based phases. This also suggests that Al at.% in bulk Ti_2_(Ga_x_Al_1−x_)C MAX is sufficient, which also indicates that improved Al activity can be significant in enhancing the protection of MAX phases against oxidation.

The phase stability analysis is an efficient determination of the most stable equilibrium state that helps to assess the resulting reaction products for a given set of reactants. We performed detailed electronic-structure, charge density, and chemical bonding (COHP) analysis to understand the change in oxidation behavior of of Ga doped Ti_2_AlC, i.e., Ti_2_(Ga_x_Al_1−x_)C MAX. Expectedly, the COHP analysis shows that covalently bonded Ti-C shows the bonding nature in (0001) basal plane compared other pairs such as Ti–Al/Ga or C–Al/Ga, which is clearly observed by their anti-bonding behavior. The anti-bonding of Ti–Al and Ti–Ga bonds makes them easier to break, however, stronger stability of Ga doped MAX phases weakens the chemical bonding of Al with Al–Ga layer. This makes the chemical activity of Al higher compared to Ga^74^, therefore, Al atoms can diffuse easily and are readily available to form a high density protective Al_2_O_3_ layers, i.e., inward oxygen flow can be slowed down and improve the oxidation resistance of MAX phases. The electronic-structure analysis shows change in interlayer interaction between Ti–C and Al/Ga as one of the reasons why Al chemical activity may increase with Ga doping. This study can be useful guideline to understand to role of alloying on electronic, thermodynamic, and oxidation related mechanisms in other MAX phases. Since the oxidation is a surface phenomenon, our study may also be helpful to understand the oxidation behavior of two-dimensional (2D) MAX phases, i.e., MXenes (transition metal carbides, carbonitrides and nitrides).

## Data Availability

The data used in the manuscript is available with corresponding author on reasonable request.
